# Development of an Effective and Cost-Saving Synergistic-Antibacterial Therapy for Prevention of Endophthalmitis

**DOI:** 10.3390/antibiotics14060588

**Published:** 2025-06-07

**Authors:** Huy Dong, Phat Tran, Keaton Luth, Dana Thalman, Coby Ray, Pamela Lin, Staci Moss, Abdul Hamood, David McCartney, Ted W. Reid

**Affiliations:** 1 Department of Ophthalmology and Visual Sciences, Texas Tech University Health Sciences Center (TTUHSC), Lubbock, TX 79430, USA; andersondongdong1991@gmail.com (H.D.); phat.tran@ttuhsc.edu (P.T.); keaton.luth@gmail.com (K.L.); dthalman@kumc.edu (D.T.); coby.ray@ttuhsc.edu (C.R.); pamelalin123@gmail.com (P.L.); stacie.moss@ttuhsc.edu (S.M.); david.mccartney@ttuhsc.edu (D.M.); 2 Department of Immunology and Molecular Microbiology, Texas Tech University Health Sciences Center, Lubbock, TX 79430, USA; abdul.hamood@ttuhsc.edu

**Keywords:** endophthalmitis, moxifloxacin, azithromycin, tobramycin, cefuroxime, cefazolin, synergisim, antimicrobials, vigamox, *Staphylococcus epidermidis*

## Abstract

Background: Endophthalmitis, associated with intraocular procedures, is an infection of the eye that can rapidly result in substantial irreversible loss of vision and may even lead to removal of the eye. Prevention strategies vary globally and often include antibiotic use—often consisting of a broad-spectrum mono-therapeutic agent. The purpose of this study is to test the efficacy and value of combinations of cefuroxime (cefu), cefazolin (cefa), azithromycin (azith), and/or tobramycin (tob) as alternatives to the use of moxifloxacin. We looked for synergism since these antimicrobials used different mechanisms of inhibition. Methods: Initially, we examined individual antimicrobials to determine the minimal bactericidal concentrations (MBC) of each individual treatment against *Klebsiella pneumonia*, *Pseudomonas aeruginosa*, *Staphylococcus aureus*, two clinical isolates of MRSA, and *Staphylococcus epidermidis*, by both the Zone of Inhibition (ZOI) and the Colony Forming Unit (CFU) assays. We then used these data in a combinatorial study. Results: We found combinations that were synergistic against all the bacteria tested, resulting in total eradication [8 logs] of all bacteria. We found that the ZOI assay provided less consistent results than the CFU assays. Conclusions: We have found combinations of these antimicrobials that were synergistic in the total eradication (8 logs) of all bacteria tested. These three combinations were: cefuroxime/azithromycin; azithromycin/tobramycin; and cefuroxime/tobramycin. Moxifloxacin (Vigamox) did not completely eradicate *Staphylococcus epidermidis.* These combinations can then be used as eye drops to serve as a prophylactic for endophthalmitis after eye injections and eye surgery.

## 1. Introduction

Cataract surgery is the most commonly performed invasive surgical operation worldwide [1,2]. There are approximately 3.3 million cataract surgeries performed annually in the USA [3]. Du et al. reported that the incidence of endophthalmitis after cataract surgery is 0.127% at 6 weeks and 0.195% at 6 months in the Medicare population [2], while other studies reported that the incidence of acute-onset postoperative endophthalmitis ranges from 0.03% to 0.2% [1,4,5,6]. Endophthalmitis is an infection of the eye that can rapidly result in substantial loss of vision and may require enucleation. Currently, fourth generation fluoroquinolones are often used preoperatively in association with cataract surgery in the US. However, the drawbacks of this treatment are the narrowing spectrum of action of this drug, the cost (~USD 2000.00/g of antibiotic in a 3 mL bottle, current source average data), and the evolution of fluoroquinolone resistance among coagulase-negative Staphylococcus endophthalmitis isolates [7,8]. It was previously reported that from 56 to 90 percent of organisms causing infection in postoperative endophthalmitis are Gram-positive, the most common of which is S. epidermidis [9]. Moreover, there is concern that late-generation fluoroquinolones may be less effective in preventing endophthalmitis caused by methicillin-resistant Staphylococcus aureus (MRSA) [10,11]. Sharifi et al. investigated the role of cost as a potential factor in choosing a specific antibiotic for the prevention of endophthalmitis, and they found that none of the fluoroquinolones were cost-saving in all scenarios modelled [12]. In contrast to the fluoroquinolones, cefuroxime (~USD 2.33/g), cefazolin (~USD 0.77/g), azithromycin (~USD 3.66/g), and tobramycin (~USD 37.50/g), are quite cost effective, at current costs [13]. Also, intracameral cefuroxime has proven to be efficacious in reducing postoperative endophthalmitis overall [14,15,16,17,18]. Similarly, Garat et al. reported that the cefazolin was effective in treatment of endophthalmitis [19]. In addition, tobramycin has proven useful in controlling both superficial and deep infections of the eye and ocular adnexa (i.e., blepharitis, conjunctivitis, keratitis, and endophthalmitis) [20,21].

We compared the effectiveness of combinations of cefuroxime, cefazolin, azithromycin, and tobramycin as possible replacements for moxifloxacin, in commercially available concentrations, against both Gram-negative and Gram-positive bacteria associated with endophthalmitis. The significance of this research is two-fold, relating to medicine and economics. Medically, it may reduce the possible emergence of antibiotic-resistant mutant strains. The development of strain-resistance to a combination of two antibiotics is far less than that of one antibiotic. Financially, an enhanced spectrum of efficacy against common ocular pathogens may reduce the incidence of endophthalmitis, thereby reducing health care costs. In addition, the cost of the generics is considerably less than the fluoroquinolones.

## 2. Results

### 2.1. Individual Antibiotic Eradication Ability

#### 2.1.1. Cefazolin

Initially, the antibiotic eradication ability was individually determined to find the minimal bactericidal concentrations (MBC) of each antibiotic, against *Klebsiella pneumonia* clinical isolate (CI), *Pseudomonas aeruginosa* PAO1 GFP, *Staphylococcus aureus* AH133 GFP, Methicillin resistant *Staphylococcus aureus* CI 121, Methicillin-resistant *Staphylococcus aureus* CI 139, and *Staphylococcus epidermidis* CI using the disc diffusion (ZOI) and the colony forming unit (CFU) assays.

**Figure 1:**
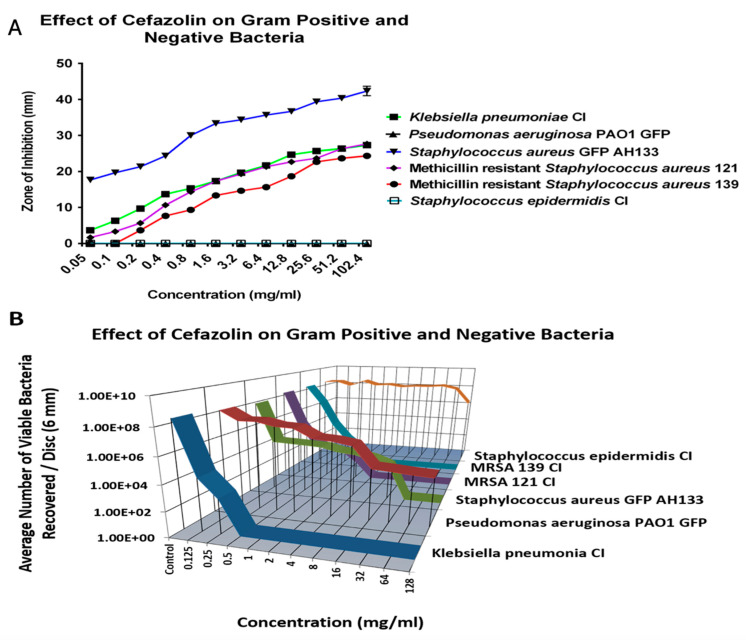
The ZOI (**A**) of Gram-positive and negative bacteria on the Cefazolin discs was measured in mm, with the diameter of the disc subtracted from total diameter of the zone; (**B**) the bacteria remaining on the Cefazolin discs was quantified by the colony forming units assay (listed as viable bacteria).

When examining Cefazolin, *Staphylococcus epidermidis* CI, and *Pseudomonas aeruginosa* PAO1 GFP, remained resistant to all tested concentrations (Figure 1A) and showed no Zone of Inhibition. The CFU assay confirmed the resistance of *S. epidermidis* CI and *P. aeruginosa* PAO1 GFP against Cefazolin. Figure 1B shows that even at the highest tested concentration of 128 mg/mL, there was an average of over six logs of both *S. epidermidis* CI and *P. aeruginosa* PAO1 GFP recovered per disc. However, at the lower concentrations, cefazolin effectively eliminated K. pneumonia CI, S. aureus AH133, and two clinical isolates of Methicillin-resistant strains, Figure 1.

#### 2.1.2. Cefuroxime

**Figure 2:**
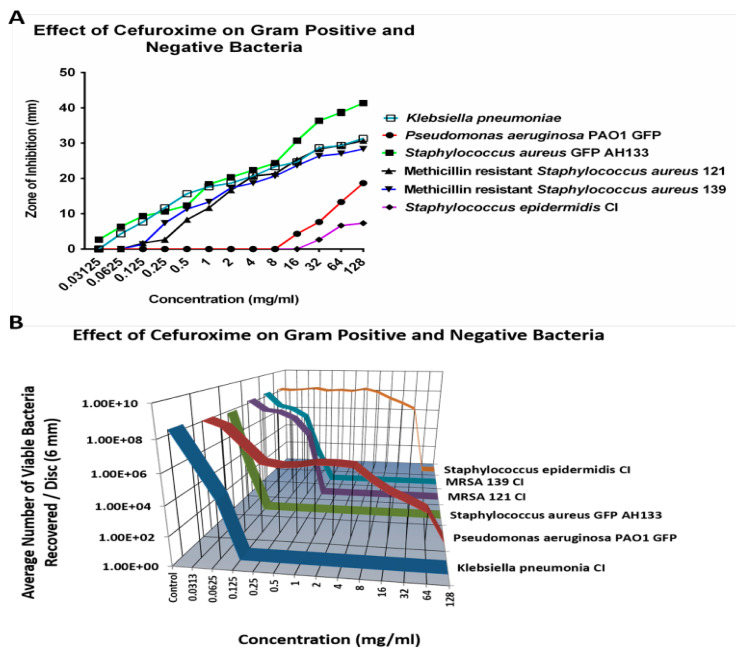
The ZOI (**A**) of Gram-positive and negative bacteria on the Cefuroxime discs were measured in mm, with the diameter of the disc subtracted from total diameter of the zone; (**B**) the bacteria remaining on the Cefuroxime discs was quantified by the CFU assay (listed as viable bacteria).

Similarly to Cefazolin, *S. epidermidis* CI and *P. aeruginosa* PAO1 GFP also remained resistant to most tested concentrations of Cefuroxime. The exception was that the 64 mg/mL concentration eradicated S. epidermidis Cl, as shown in Figure 2B. Figure 2A shows there is an increase in the size of the ZOI for both *S. epidermidis* CI and *P. aeruginosa* PAO1 GFP bacteria at 16 mg/mL of Cefuroxime, but the CFU assay showed that there are an average of over six logs and four logs of bacteria recovered per disc for *S. epidermidis* CI and for *P. aeruginosa* PAO1 GFP, respectively.

#### 2.1.3. Azithromycin

**Figure 3:**
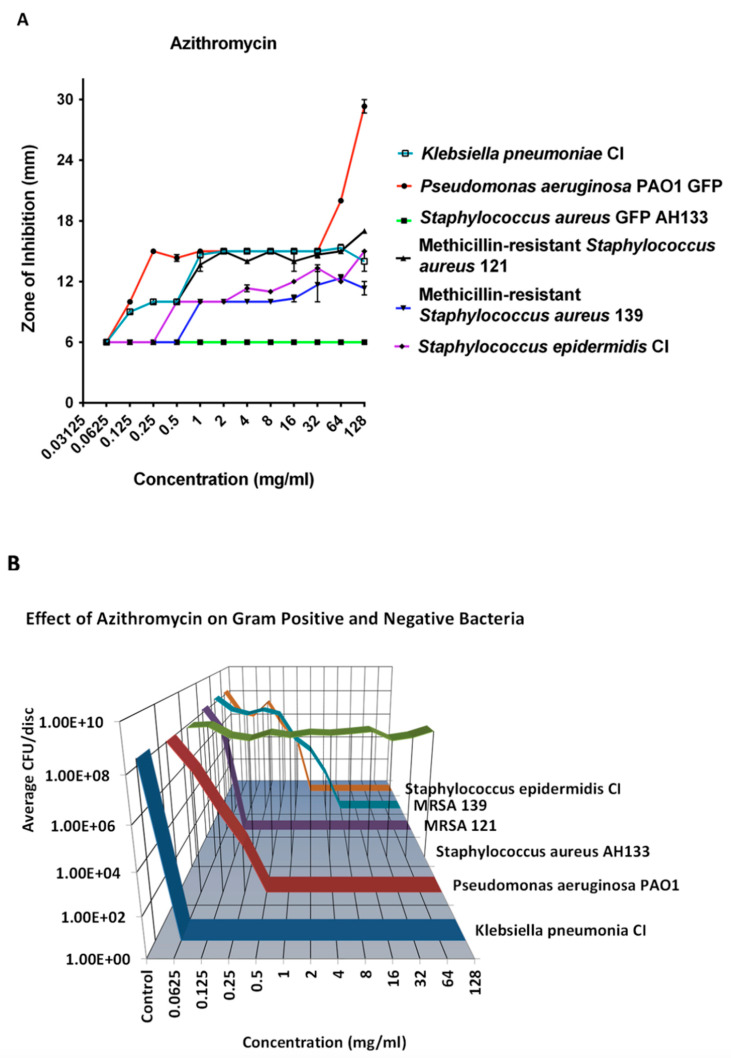
The ZOI (**A**) of Gram-positive and negative bacteria on the Azithromycin discs was measured in mm, with the diameter of the disc subtracted from total diameter of the zone); (**B**) the bacteria remaining on the Azithromycin discs was quantified by the CFU assay.

Azithromycin was effective in the eradication of 8 logs of all the bacteria tested, except *S. aureus* at 8 mg/mL (Figure 3B). It did not eradicate *S. aureus* at even higher concentrations, while eradicating two strains of MRSA. This is seen in both the ZOI study (Figure 3A) and the CFU determination (Figure 3B).

#### 2.1.4. Tobramycin

**Figure 4:**
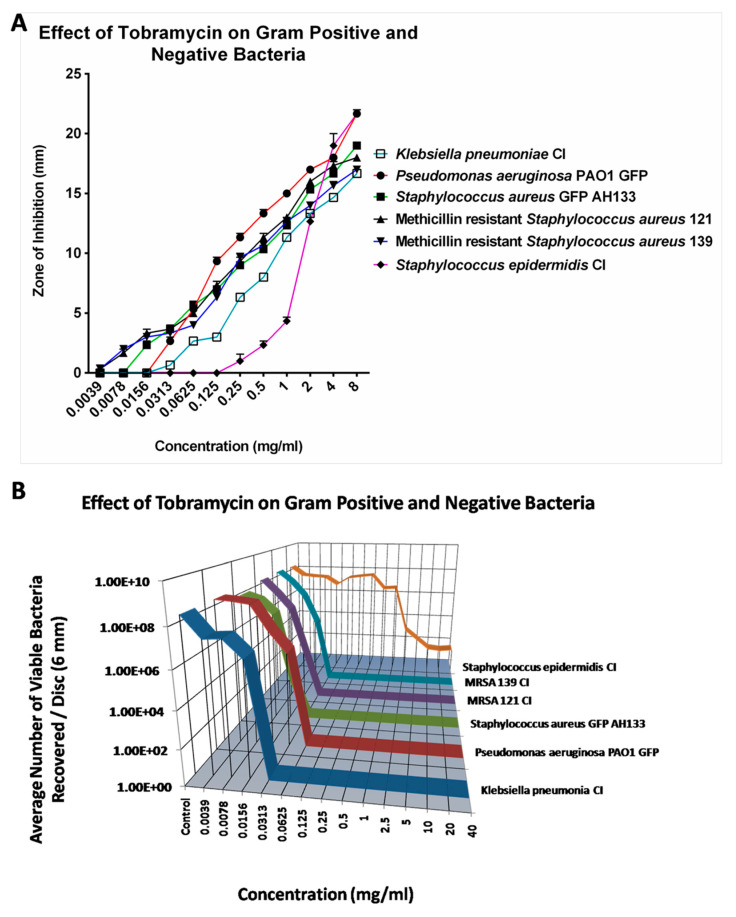
The ZOI (**A**) of Gram-positive and negative bacteria on the Tobramycin discs was measured in mm, with the diameter of the disc subtracted from total diameter of the zone; (**B**) the bacteria remaining on the Tobramycin discs was quantified by the CFU Assay (listed as viable bacteria).

Compared to Azithromycin, Cefazolin and Cefuroxime, Tobramycin is the most effective antibiotic against all six tested bacteria. The Zone of Inhibition for most bacteria increases as Tobramycin is increased from 0.0039 mg/mL to 8 mg/mL. However, as shown in Figure 4A, Tobramycin did not show ZOI against *S. epidermidis* CI until the concentration reached 1.0 mg/mL. Similar results were found by CFU assay as shown in Figure 4B. The CFU assay showed that 0.125 mg/mL of Tobramycin effectively eliminated most bacteria except *S. epidermidis* CI. *S. epidermidis* CI remained resistant to Tobramycin up to 1 mg/mL of Tobramycin; however, Tobramycin never completely eradicated the *S. epidermidis,* even at 40 mg/mL.

**Figure 5:**
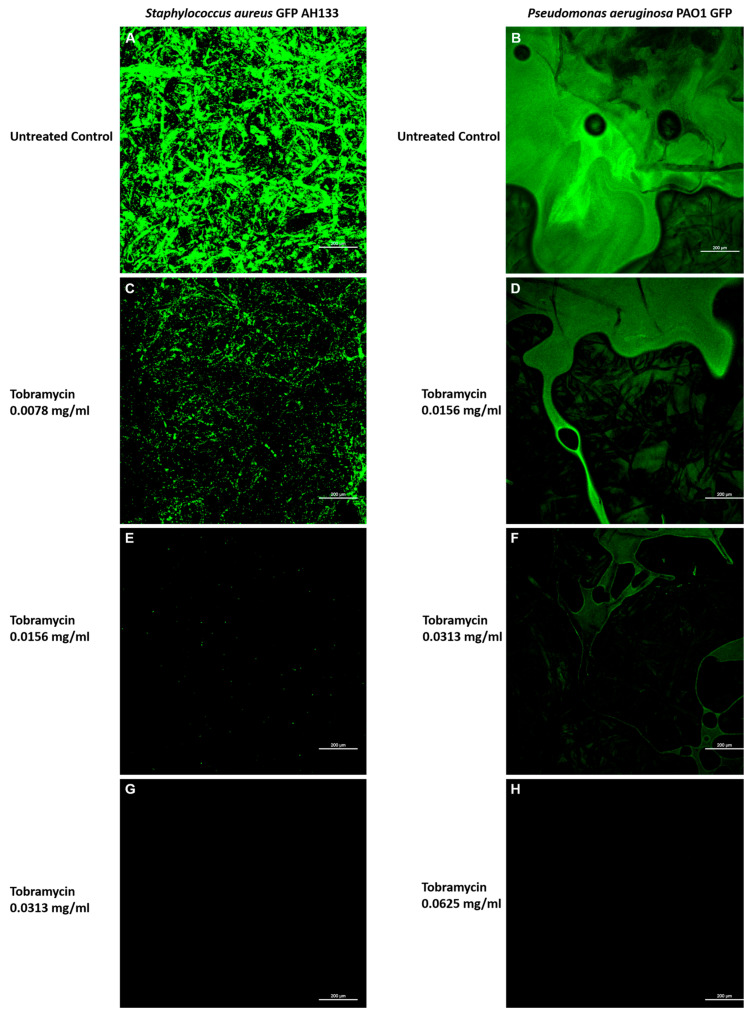
Confocal Laser Scanning Microscopy images of the *S. aureus* GFP AH133 (**A**,**C**,**E**,**G**), and the *P. aeruginosa* PAO1 GFP (**B**,**D**,**F**,**H**), that remained on the control and Tobramycin discs. Bar Scale is equal to 200 µm.

The effects of Tobramycin alone can also be seen visually in Figure 5, when observed by Confocal Laser Scanning Microscopy, showing images of the *S. aureus* GFP AH133 (Figure 5A,C,E,G), and the *P. aeruginosa* PAO1 GFP (Figure 5B,D,F,H), that remained on the control and Tobramycin discs. Bar Scale is equal to 200 µm.

#### 2.1.5. Moxifloxacin and Gatifloxacin

**Figure 6:**
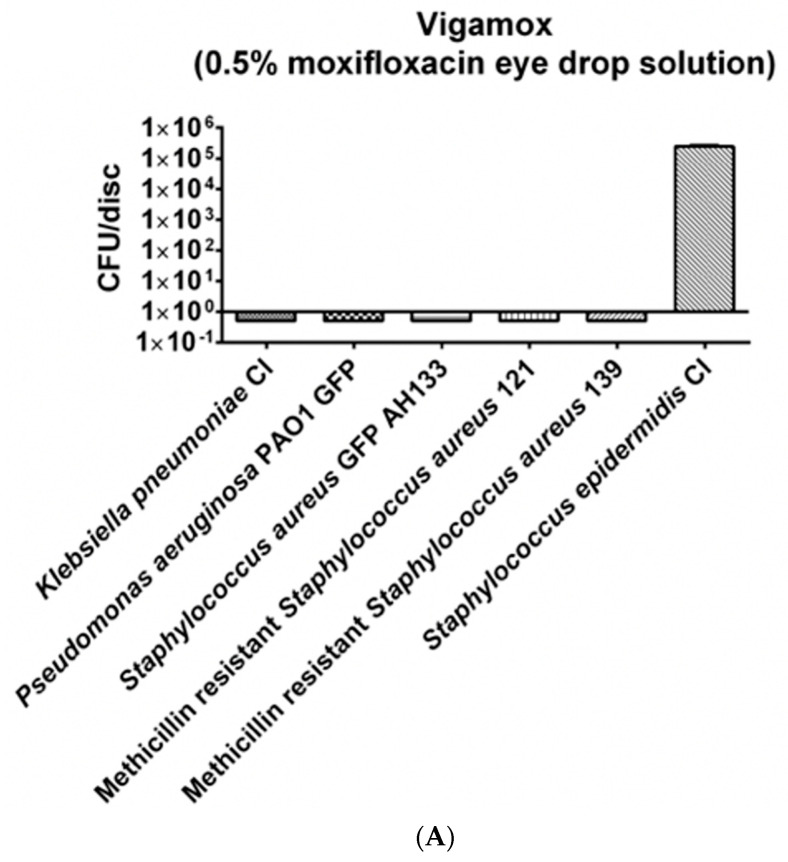

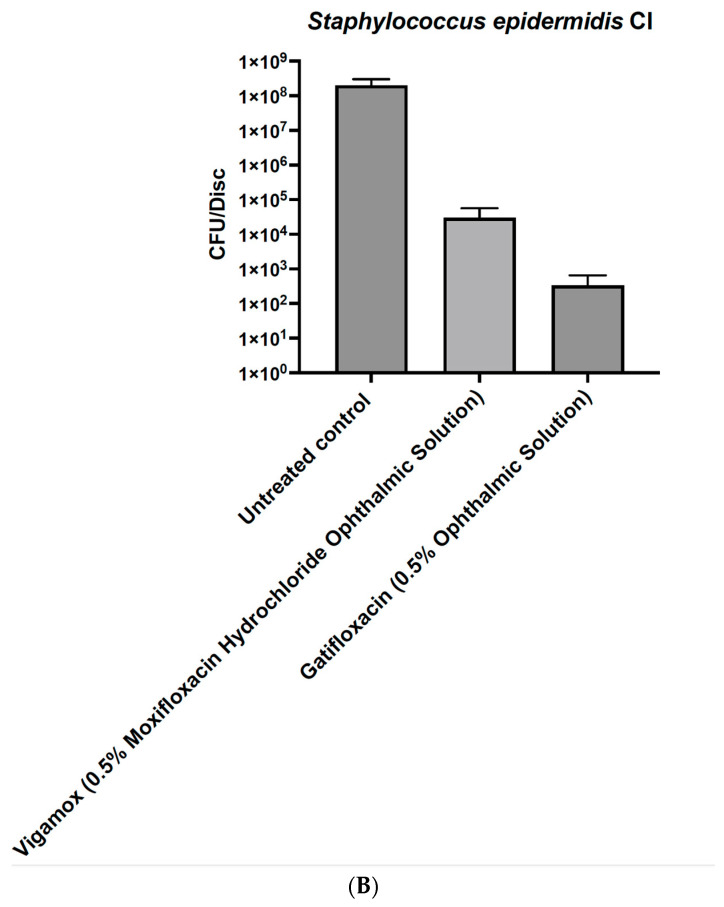
(**A**) The bacteria remaining on the Moxifloxacin hydrochloride discs was quantified by the CFU Assay. (**B**) Comparison of the *Staphylococcus epiderdis Cl* remaining on the Moxifloxacin hydrochlorideand Gatifloxacin discs was quantified by the CFU Assay.

The disc diffusion (ZOI) and colony forming unit (CFU) assays were also performed with Moxifloxacin and Gatifloxacin; these drugs are currently used in the treatment and prevention of endophthalmitis. As shown in Figure 6A, Moxifloxacin effectively eliminated *Klebsiella pneumonia* CI, *Pseudomonas aeruginosa* PAO1 GFP, *Staphylococcus aureus* GFP AH133, and two clinical isolates of Methicillin resistant strains, but it failed to eliminate *S. epidermidis* CI. We then teste another member of the quinolone antibiotics, Gadtifloxacin, and it too failed to eliminate *S. epidermidis* CI, as seen in Figure 6B.

### 2.2. Combinations of Antimicrobials

We selected concentrations of individual antibiotics and used these concentrations in an antibiotic-combination study. The results allowed us to examine whether these antibiotic combinations are additive, synergistic, or antagonistic, when compared to their individual concentrations.

Combinations were tested by decreasing the concentration of the individual antibiotic in the combinations by a factor of two. This was used to find the minimum concentration of the antibiotic combinations that eliminated all six tested bacteria.

#### 2.2.1. Cefuroxime and Azithromycin

**Figure 7:**
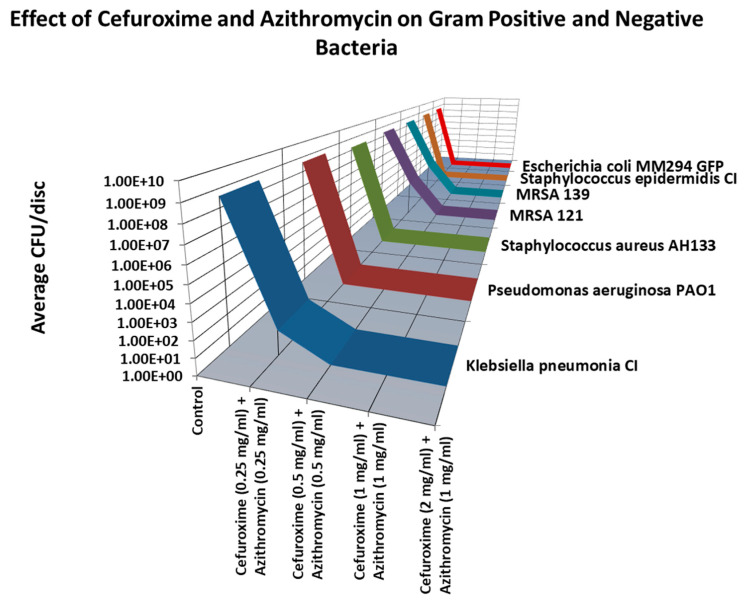
Cefuroxime and Azithromycin combination discs were quantified by the colony forming units assay. Total eradication (8 logs) of all bacteria tested were seen at 0.5mg/mL Cefuroxime and Azithromycin 0.5 mg/mL.

As shown in Figure 7, the lowest combination of Cefuroxime and Azithromycin that eradicated all (8 logs) of the bacteria tested was Cefuroxime (0.5 mg/ml) and Azithromycin (0.5 mg/mL).

#### 2.2.2. Cefuroxime/Tobramycin or Azithromycin/Tobramycin

Experiments similar to those applied to Cefuroxime and Azithromycin were carried out with Cefuroxime/Tobramycin and Azithromycin/Tobramycin combinations. The lowest combinations eradicating all 8 logs of bacteria are seen in Table 1.

#### 2.2.3. Cefazolin

**Figure 8:**
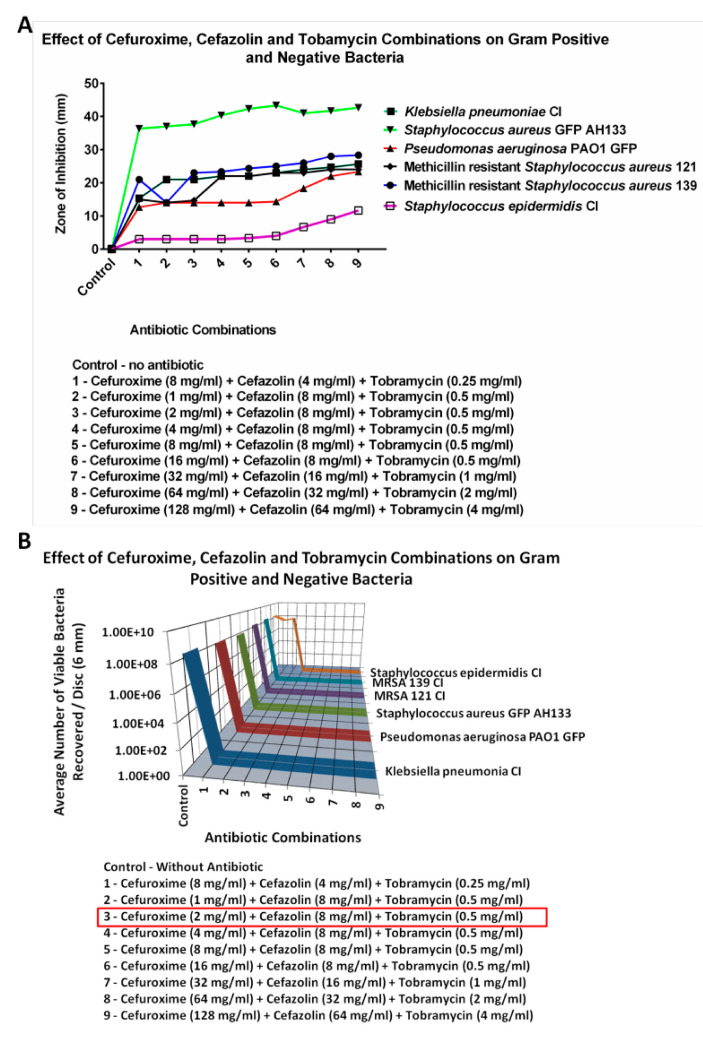
The ZOI (**A**) of Gram-positive and negative bacteria on the Tobramycin, Cefazolin, and Cefuroxime combination discs was measured in mm, with the diameter of the disc subtracted from total diameter of the zone; (**B**) the bacteria remaining on the Tobramycin, Cefazolin, and Cefuroxime combination discs was quantified by the CFU Assay (listed as viable bacteria). The concentrations in the red box are the lowest concentrations which irradicated all the bacteria tested.

The lowest concentrations for Cefazolin that would eradicate all 8 logs of the bacteria tested had to be carried out in a triple combination. These results are seen in Figure 8. The concentrations of individual antibiotics in the combination that eliminated all six bacteria consisted of 2 mg/mL of Cefuroxime, 8 mg/mL of Cefazolin and 0.5 mg/mL of Tobramycin. In a triple combination of Azithromycin, Cefuroxime and Tobramycin it was found that the lowest combination where all the bacteria were eradicated (8 logs) was Azithromycin 0.25 mg/mL, Cefuroxime 0.25 mg/mL and Tobramycin 0.312 mg/mL.

#### 2.2.4. Zone of Inhibition Results

With each of the antibiotics, the ZOI assay was not as sensitive to changes in the inhibition of *P. aeruginosa* and *S. epidermidis* as the CFU assay.

## 3. Discussion

The results showed that the double antibiotic combinations of Azithromycin, Cefuroxime and Tobramycin was synergistic and bactericidal against all Gram-negative and positive organisms tested. This is important because acute postoperative endophthalmitis has been shown to be caused by a broad spectrum of bacteria [9]. Due to the economy of using antibiotic combinations which are off patent, they can be used to replace cheap materials such as betadine (Povidone) which we have shown to be very ineffective [22].

A comparison of these double antibiotic combinations versus Vigamox (0.5% Moxifloxacin) found that Vigamox (0.5% Moxifloxacin) exhibited only 3–4 logs of inhibition of *Staphylococcus epidermidis* CI., and Gatifloxacin inhibited 6 logs, while the different double combinations of Azithromycin, Cefuroxime and Tobramycin completely eliminated *Staphylococcus epidermidis CI* (100% inhibition; Table 1).

The resistance of *Staphylococcus epidermidis* CI against Vigamox (0.5% Moxifloxacin), a third-generation fluoroquinolone, was confirmed by a previous study performed by Schimel AM [8]. Over 21.5 years of study, 168 patients were identified as having culture-proven endophthalmitis caused by coagulase-negative *staphylococcus* [8]. In addition, despite the evolving mechanism of different generations of fluoroquinolones, the frequency of resistance to coagulase-negative staphylococcus continues to increase [8].

The triple antibiotic combination of Cefazolin, Cefuroxime and Tobramycin, was also highly effective at eliminating *Klebsiella pneumonia* clinical isolate (CI), *Pseudomonas aeruginosa* PAO1 GFP, *Staphylococcus aureus* AH133 GFP, Methicillin resistant *Staphylococcus aureus* CI 121, Methicillin-resistant *Staphylococcus aureus* CI 139, and *Staphylococcus epidermidis* CI. The Zone of inhibition (ZOI) and CFU assays showed no growth for these six Gram-negative and positive bacteria.

A comparison of the different combinations can be seen in Table 1. For topical ocular application, an important difference between our antibiotic combination solutions and Vigamox (0.5% Moxifloxacin) is that the combination was effective at much lower concentrations than when used as monotherapy. Cefuroxime worked at 2 mg/mL with Azithromycin or Tobramycin, while alone it is typically used topically at 25–100 mg/mL. Cefazolin worked at 8 mg/mL (triple combination), while alone it is used at 25–100 mg/mL. Tobramycin worked at 0.5 mg/mL with Azithromycin or Cefuroxime, while alone it is used at 14 mg/mL. Azithromycin was effective at 1 mg/mL in combination with Cefuroxime or Tobramycin, while alone it is used at 10 mg/mL. When Cefuroxime’s concentration was dropped to 1 mg/mL, there was no activity of the combination against *S. epidermidis* CI.

## 4. Materials and Methods

### 4.1. Bacterial Strains, Media, and Growth Conditions

The laboratory strains of bacteria tested were *S. aureus* GFP AH133 and *P. aeruginosa* PAO1GFP strains, both of which constitutively express green fluorescent protein from plasmids pCM11 and pMRP9-1, respectively [23,24]. The strains were routinely grown in Luria–Bertani (LB) broth at 37 °C with shaking (250 rpm). To maintain pCM11 in AH133, LB was supplemented with 1 μg/mL erythromycin. To maintain pMRP9-1 in PAO1, LB broth was supplemented with 300 μg/mL carbenicillin. The clinical isolates studied were *S. epidermidis*, *K. pneumoniae*, and two strains of *S. aureus*, which are methicillin-resistant (MRSA).

The efficacy of individual antibiotic solution was examined using LB broth medium (#113002022, MP Biomedical, Solon, OH, USA) and LB-Agar medium (#113002222, MP Biomedical, Solon, OH, USA) as the growth medium. The clinical isolates were obtained from the Clinical lab at Texas Tech University Health Sciences Center under an approved Institutional Review Board protocol, Texas Tech University Medical Center/Lubbock, TX, USA.

### 4.2. Antibiotic Solution Preparation

Tobramycin (#NDC 63323-306-02), cefuroxime (#NDC 25021-118-10), and cefazolin (#NDC 0409-2585-01) were purchased from Cardinal Health, Dublin, OH, USA. Azithromycin (#NDC 55150-174-10) was purchased from Auromedics, East Winsor, NJ, USA. 5% Moxifloxacin hydrochloride (Vigamox) ophthalmic solution (#NDC 0065-4013-03) was purchased from Alcon, Fort Worth, TX, USA. Antibiotics were prepared in sterile dH_2_O into several stock solutions from the same batch following the manufacturer’s recommendations. For consistency, antibiotic solutions were prepared for each test from stock solutions stored at the manufacturer’s temperature recommendations. For each experiment, antibiotic working solutions were made fresh on the day of inoculation. The range of antibiotic concentrations used for determining MICs were prepared in doubling dilution steps up and down from 1 mg/mL.

### 4.3. Disc Diffusion Assay

The disc diffusion testing method (Zone of Inhibition, ZOI), as previously described, was used for all experiments [25]. Analysis of any remaining bacteria on the antibiotic discs was conducted using the colony forming (CFU) assay. The disc diffusion testing method (ZOI) and CFU assay are standardized, reliable susceptibility testing techniques. Bacteria were grown overnight in LB medium. The following day, the bacterial culture was washed in Mueller Hinton (MH) broth (#70192, Sigma-Aldrich, St. Louis, MO, USA), and the bacterial suspension was adjusted to an OD_600_ of 0.1 (which is equivalent to the 0.5 McFarland standard of ~1 × 10^7^ bacterial cell/mL) in MH broth,, according to the standard guidelines of the National Committee for Clinical Laboratory Standards [25]. Following this, a sterile cotton swab was dipped into the adjusted bacterial culture, and a lawn of bacteria was made on an LB Agar plate using the dipped swab. The antibiotic discs were prepared by adding 20 μL of antibiotic solution onto 6 mm diameter blank BD BBL Sensi-Disc Antimicrobial Susceptibility Test Discs (#B31039, Fisher Scientific, Waltham, MA, USA).

Three antibiotic discs were distributed evenly onto an LB Agar surface. Three separate plates were measured; thus, nine points were determined for each bacterial strain. The plates were then incubated at 37 °C for 24 h before the results were read and recorded. The diameters of the zones of complete and clear inhibition, including the diameter of the disc, were measured to the nearest millimetre with a ruler; however, the diameter of the disc was subtracted from all the measurements in the calculation and graphing

### 4.4. Confocal Laser Scanning Microscopy (CLSM) of the GFP Containing Bacteria

In addition to the disc-diffusion study above, the discs from the *S. aureus* GFPAH133/pCM11 and *P. aeruginosa* GFPPAO1/pMRP9-1 plates were examined under the CLSM. *S. aureus* GFP and *Pseudomonas aeruginosa* GFP are lab strains, which constitutively express green fluorescent protein from plasmids pCM11 orpMRP9-1 when grown in the presence of 1 μg/mL erythromycin or 300 μg/mL carbenicillin, respectively. Images were captured using the CLSM, and comparison was made between the different concentrations of antibiotic and the control. Visualization of the *S. aureus* GFP AH133 and *P. aeruginosa* PAO1 GFP bacteria was accomplished with a Nikon Eclipse Ti upright confocal laser scanning microscope (Nikon, Melville, NY, USA). Samples were examined under 10× objective lens, FITC fluorescence to eyes laser (488.0 nm), 4 channel confocal, and 512 scan size. The images were processed and analyzed using NIS-Elements AR Imaging Software 16.97.2.

### 4.5. Determination of Colony Forming Units

All strains of the microorganisms remaining on the discs, following the disc-diffusion assay, were quantified by the CFU assay as previously described [26]. Following incubation, each disc piece was transferred to a sterile 1.5 mL micro centrifuge tube containing 1 mL of PBS (pH = 7.4) for enumeration of bacteria. The tubes were placed in a water bath sonicator for 10 min to loosen the cells within the disc and then vigorously vortexed 3 times for 1 min to detach the cells. One hundred μL was removed from the 1 mL suspension of cells and was serially diluted (10-fold) in PBS, and 10 μL aliquots of each dilution were spotted onto LB Agar plates. In addition, the remaining 900 μL of the 1 mL zero dilution sample was also plated on a different LB Agar plate to look for any cells in case none were detected from the serial dilution. The equation used to calculate the count was the recovered number of colonies × dilution factor/inoculums size in mL. This means that if only one bacterial cell was originally in the tube, there would be a 90% chance of detecting it in the 900 μL. All experiments were performed in triplicate on each plate, and all measurements were repeated on three separate plates. Thus, nine measurements in total were carried out on each concentration.

### 4.6. Statistical Analyses

Results of the colony forming unit assays (CFU) were statistically analyzed using GraphPad InStat 3.06 (GraphPad Software, San Diego, CA, USA). Significance between pairs of values—control versus one treatment group—was calculated using an unpaired two-tailed t test when SD was not significantly different and when a Gaussian distribution was observed. If SD was significantly different, the Welch correction was applied to the un-paired two-tailed t test. When non-Gaussian distribution was observed (Kolmogorov–Smirnov test), significance was calculated by a non-parametric Mann–Whitney test. Two treatment groups were compared using the Kruskal–Wallis test (nonparametric ANOVA) with Tukey’s multiple comparisons test applied for significance. Differences were considered significant when the *p*-value was ≤0.05.

## 5. Conclusions

The results of this study may be applicable to cataract surgery and injections, as well as for bacterial keratitis. The study showed that the combination of Cefuroxime, Azithromycin, or Tobramycin showed a synergistic effect and were superior to the individual antibiotics. In addition, this combination completely eradicated (8 logs, 100%) all of the tested bacteria. These different bacteria represent approximately 70% of the bacterial eye infections in the USA and 64% of those found in the world in 2023 [27]. They included laboratory strains of *Staphylococcus aureus* and *Pseudomonas aeruginosa* and clinical isolates of *Klebsiella pneumoniae*, *Staphylococcus epidermidis*, and MRSAs. Moxifloxacin and Gatifloxacin failed to eradicate the tested clinical isolate of *Staphylococcus epidermidis.* Cefazolin had to be used in a triple combination to be effective. The combination of tested antibiotics showed enhanced efficacy, as well as enhanced value over that of Moxifloxacin and Gatifloxacin.

## Figures and Tables

**Table 1:** Concentrations in mg/mL, which in a combination of antibiotics eradicated all (8 logs) of tested bacteria.

Cefuroxime	Cefazolin	Tobramycin	Azithromycin
1.0		1.25	
0.25		0.312	0.25
2.0			1.0
		0.312	1.0
2.0	8.0	0.5	

## Data Availability

Data supporting results can be found archived in the TTUHSC Medical School Library.

## Abbreviations

The following abbreviations are used in this manuscript: MBC: minimal bacterial concentration; CFU: colony forming unit; MRSA: methicillin-resistant *Staphylococcus aureus*; GFP: green fluorescent protein; LB: Luria–Bertani; NDC: National Drug Code; MH: Mueller Hinton; ANOVA: analysis of variance; PBS: phosphate-buffered saline; SD: standard deviation; CI: clinical isolate; ZOI: Zone of Inhibition; CFU: colony forming units.
